# Macroscopic Hematuria and a Bladder Mass: Eosinophilic Cystitis in a 7-Year-Old Boy

**DOI:** 10.1155/2016/9346218

**Published:** 2016-05-31

**Authors:** Stine Bjerrum Runge, Søren Høyer, Louise Winding

**Affiliations:** ^1^Department of Radiology, Lillebaelt Hospital, Skovvangen 2-8, 6000 Kolding, Denmark; ^2^Institute of Pathology, Aarhus University Hospital, 8000 Aarhus C, Denmark; ^3^Pediatric Department, Lillebaelt Hospital, 6000 Kolding, Denmark

## Abstract

We report a case of eosinophilic cystitis in a 7-year-old boy with a history of atopic symptoms, with focus on the radiological findings. He presented with hematuria and dysuria and ultrasonography (US) showed irregular bladder wall thickening resembling a bladder mass. CT urography did not characterize the lesion any further and showed no local or distant spread. Biopsies revealed eosinophilic cystitis, a benign inflammatory condition. We found that US characterized the lesion at least as well as CT and should be the first choice of imaging. When staging is considered before biopsy, MRI should be preferred to CT. There are no specific radiological signs of eosinophilic cystitis. On follow-up, US was a safe, cost-effective imaging modality, but findings should be interpreted in a clinical context. In a child with hematuria and a bladder mass, eosinophilic cystitis is a relevant but rare differential diagnosis, especially when there is a known atopic history.

## 1. Introduction

The aim of this paper is to draw attention to the presenting symptoms and the radiologic evaluation of eosinophilic cystitis in childhood. Eosinophilic cystitis is an inflammatory condition characterized by eosinophilic infiltration of the bladder wall. It is rare amongst children with only 59 cases in the literature to date [1–5]. In a review of 54 cases, the mean age of presentation was 6.7 years, with an overweight of male cases [1]. The etiology is unknown [6, 7]. A tumor-like presentation on imaging has been reported previously, and the condition therefore requires thorough diagnostics to rule out malignant disease. We present a typical case of this atypical disease with focus on the radiological aspects of diagnosis and follow-up.

## 2. Case Presentation

A 7-year-old boy was referred to a secondary pediatric centre with gross hematuria and dysuria.

His previous medical history included atopic symptoms (wheeze, rhinitis, throat clearing, and cough), but, despite diagnostic evaluation, no asthma or allergies had been diagnosed. Physical examination revealed mild tenderness to palpation over the bladder. Urinalysis was positive for blood and protein. Blood creatinine was elevated to 56 *μ*mol/L (26–49 *μ*mol/L); infection parameters were normal. It was suspected that he suffered from urinary tract infection and antibiotic treatment was initiated, but the urine culture turned out to be negative. He had elevated IgE 665 ie (<400 ie) and slight eosinophilia in the blood 0.84 10^9^/L (<50 10^9^/L). All other hematologic parameters were normal: hemoglobin 7.8 mmol/L (6.5–8.9 mmol/L), white blood cell count 8.5 10^9^/L (4.5–12.5 10^9^/L), neutrophils 4.43 10^9^/L (1.8–8.9 10^9^/L), lymphocytes 2.5 10^9^/L (1.0–4.7 10^9^/L), and platelets 337 10^9^/L (165–435 10^9^/L).

**Figure 1:**
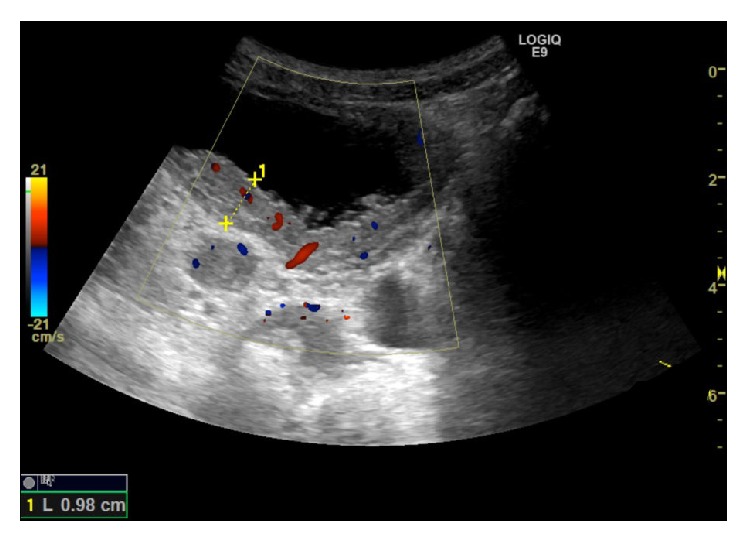
Ultrasound showed irregular bladder wall thickening of up to 1 cm resembling a tumor and Doppler flow within the wall.

**Figure 2:**
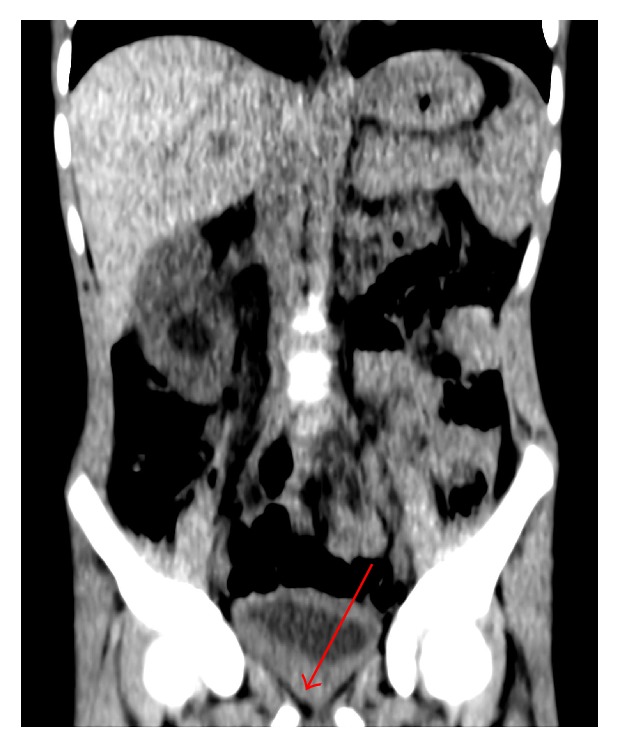
CT confirmed the finding of irregular bladder wall thickening but did not characterize the bladder lesion as well as US. Coronal reconstruction with 8 mm slice thickness and digital optimization.

One week later, he was readmitted due to recurrent terminal hematuria. Ultrasonography (US) showed right-sided hydronephrosis and hydroureter with distal occlusion by thickened ureter wall. Extending from the right ureter ostium, there was very irregular thickening of the posterior bladder wall, up to 1 cm, and Doppler flow within the wall (Figure 1). US diagnosis was infection sequelae or tumor. An acute CT urography was made to further characterize the changes in the right kidney and ureter. It confirmed the findings of the ultrasound (Figure 2) and showed contrast retention in the right kidney and pelvis but did not characterize the lesions further.

He was then transferred to a tertiary centre. A cystoscopy showed edematous mucosa with bullous excrescences from the trigonum to the right ostium and findings were very suspicious of neoplasia. A JJ catheter was inserted.

**Figure 3:**
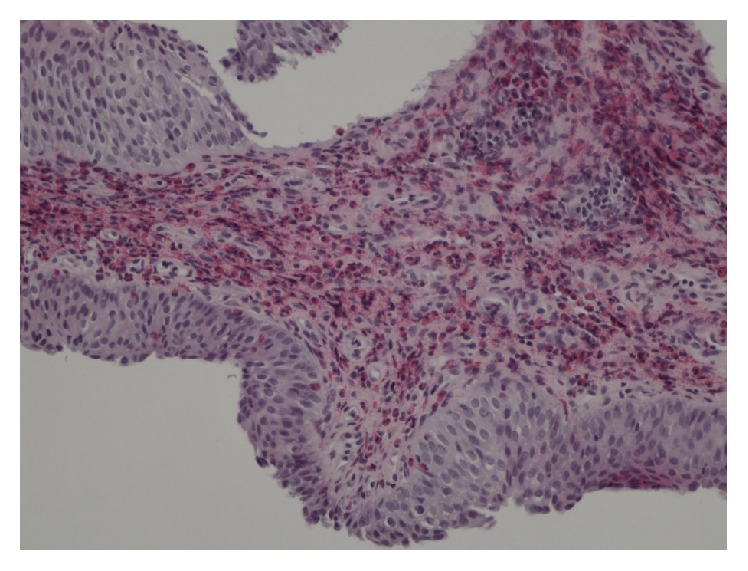
A biopsy from the bladder mucosa showing pronounced eosinophilia with free stomal granules (×200).

The biopsies showed eosinophilic cystitis and no sign of malignancy (Figure 3).

**Figure 4:**
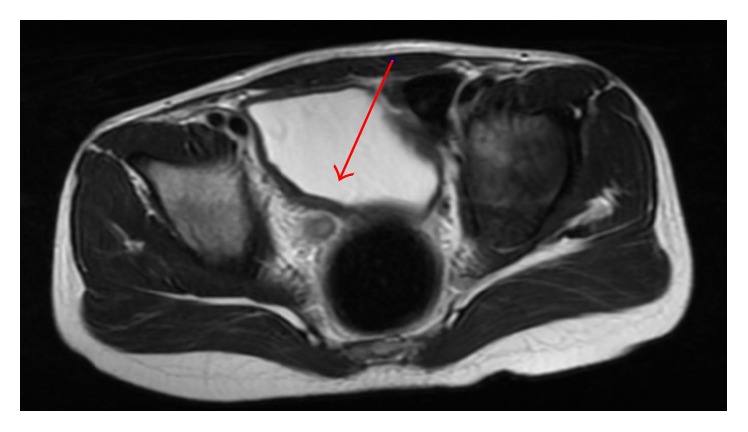
Axial T2-weighted MRI after one month showed discrete bladder wall thickening with homogenous low signal intensity (arrow).

The patient was treated empirically with antihistamine (Desloratadine 5 mg bd) and NSAID (Naproxen 5 mg/kg bd) and recovered clinically and paraclinically within 6–8 weeks. Before that he was treated with anticholinergica (Oxybutynin 2.5 mg bd) without effect. MRI after one month showed thickening of the right ureter wall and discrete bladder wall thickening. T2-weighted sequences showed homogeneous low signal intensity of the bladder wall (Figure 4).

US and cystoscopy after 3 months showed marked regression and biopsies showed nonspecific reactive changes. At removal of the JJ catheter after 7 months, the bladder looked near normal at cystoscopy. US showed slight irregular bladder wall thickening. One year later, US showed low bladder volume; thus, wall thickness could not be evaluated.

**Figure 5:**
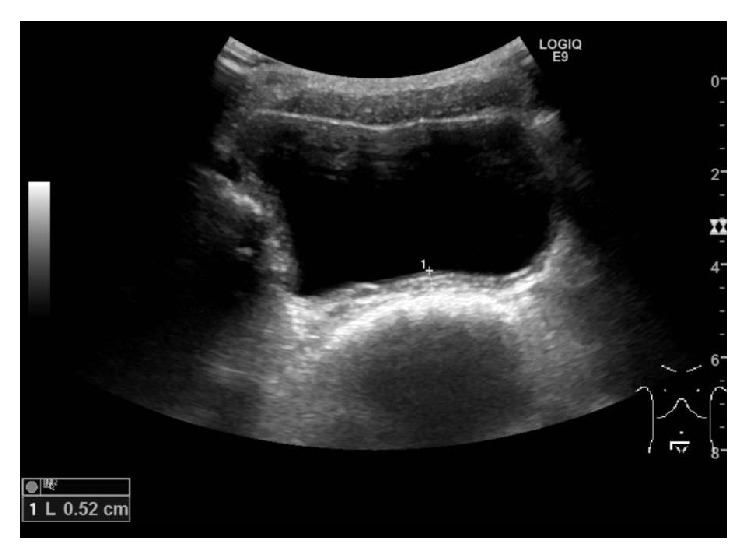
Ultrasound at clinical recurrence showed thickening of the bladder wall of up to 5 mm.

The patient was rereferred at the age of 9 years with gross hematuria and dysuria, frequent throat clearing, epigastric stomach pain, and acid reflux. The eosinophilic blood cell count was normal and US showed slight thickening of the bladder wall of up to 5 mm (Figure 5). Treatment with antihistamine for 6 weeks eliminated all symptoms. After 8 weeks, US showed persisting slight bladder wall thickening of up to 3 mm.

## 3. Discussion

Our patient presented with macroscopic hematuria and symptoms typical of cystitis. Urinalysis was not consistent with urinary tract infection, though. This fact should lead to the consideration of other diagnoses. Eosinophilic cystitis, though rare, is a relevant differential diagnosis, especially in a patient with a known atopic history. Macroscopic hematuria in a child can be a symptom of severe renal or urinary tract disease, and immediate evaluation is recommended by the Danish Pediatric Society [8]. The evaluation includes US of the kidneys and urinary tract, which in our case showed bladder wall thickening resembling a bladder mass.

A pseudotumoral appearance of (hemorrhagic) cystitis has been described in viral cystitis, being caused by chemotherapy or indwelling catheters, granulomatous cystitis, inflammatory myofibroblastic tumor, and eosinophilic cystitis [1, 9, 10].

Benign bladder lesions in children include several rare entities such as fibromas, hemangiomas, schwannomas, hematomas, leiomyomas, endometriomas, and transitional cell papillomas.

Malignant lesions include rhabdomyosarcomas (RMS), leiomyosarcomas, urothelial carcinomas, and secondary involvement such as lymphomas, with RMS being by far the most common [9, 11, 12].

In a study of 65 pediatric patients with polyps and masses in the urinary bladder, reactive/inflammatory masses comprised 20 (31%), RMS comprised 15 (23%), and other neoplasms (benign and malignant) 22 (33%) [12].

Sonographically, eosinophilic cystitis can present as concentric bladder wall thickening or a more focal, tumor-like appearance as in our case. Hydroureteronephrosis was seen in other cases too [1, 5].

To our knowledge, no specific US signs of eosinophilic cystitis have been reported.

Rosenberg et al. reported 17 children with benign cystitis in whom imaging mimicked RMS. They concluded that sonographic findings of isoechoic bladder wall thickening (focal, multifocal, or circumferential distribution), intact mucosa, and bullous lesions strongly suggested inflammation and not malignancy. Also, changing mass contour and thickness with increasing bladder filling should be signs of inflammatory thickening [13].

RMS has a highly variable appearance on US, but polypoid projection into the bladder lumen makes this diagnosis more likely [1, 9].

Color Doppler US can be useful, as some differential diagnoses such as hematomas can be excluded if Doppler flow is missing.

In our case, CT did not characterize the bladder lesion any further. As RMS is a potential and common differential diagnosis, however, a CT scan can be useful to assess any local or distant spread (staging). This is also well examined with MRI though [1], and when the setting and logistics allow for it, we recommend that MRI should be preferred in the pediatric population to avoid potentially harmful radiation exposure (following the ALARA principle). In our case, cystoscopy and biopsy could well have been the next step after initial ultrasound, but if a lesion turns out to be malignant, biopsy potentially could have led to seeding of tumor cells. The relevance of the follow-up cystoscopy performed can be discussed.

Tamai et al. report a case of eosinophilic cystitis in an 8-year-old girl where MRI showed distinct low signal intensity on T2-weighted images, which was suggested to represent high cellularity due to massive eosinophilic infiltration [14]. Eosinophilic cystitis has been reported isointense on T2-weighted sequences in the adult population [15]. In our case, MRI was used in early follow-up and gave a clear impression of the remaining extent of the disease.

The normal bladder wall thickness in children depends on bladder volume. A normal bladder wall thickness of up to 1.5–3 mm (full bladder) has been found [16, 17]. Our patient had continuing bladder wall thickening on follow-up US. This could be due to chronic postinflammatory changes after the initial episode, consistent with biopsy findings of nonspecific reactive changes after 3 months. The significance of bladder wall thickening found by US at the time of clinical recurrence thus remains unclear and treatment relied on clinical presentation. Indeed, slight bladder wall thickening persisted despite clear effect on symptoms.

## 4. Conclusion

In a child with hematuria and clinical cystitis, but sterile urine, eosinophilic cystitis is a relevant but rare differential diagnosis, especially when there is a known atopic history. US should be the first choice of imaging and MRI should be preferred to CT when staging is considered before biopsy. Eosinophilic cystitis can present as a bladder tumor radiologically and macroscopically and since there are no specific radiological signs, biopsy remains required. US is a safe, cost-effective imaging modality on follow-up, but findings should be interpreted in a clinical context.
